# Therapeutic drug monitoring of mycophenolic acid (MPA) using volumetric absorptive microsampling (VAMS) in pediatric renal transplant recipients: ultra-high-performance liquid chromatography-tandem mass spectrometry analytical method development, cross-validation, and clinical application

**DOI:** 10.1007/s43440-023-00509-w

**Published:** 2023-07-15

**Authors:** Arkadiusz Kocur, Jacek Rubik, Paweł Czarnowski, Agnieszka Czajkowska, Dorota Marszałek, Maciej Sierakowski, Marta Górska, Tomasz Pawiński

**Affiliations:** 1 Department of Drug Chemistry, Medical University of Warsaw, 1 Banacha St, 02-091 Warsaw, Poland; 2 Pharmacokinetics Laboratory, Department of Biochemistry, Radioimmunology, and Experimental Medicine, The Children’s Memorial Health Institute, Dzieci Polskich 20, 04-730 Warsaw, Poland; 3 Department of Nephrology, Kidney Transplantation, and Arterial Hypertension, The Children’s Memorial Health Institute, Dzieci Polskich 20, 04-730 Warsaw, Poland; 4 Department of Genetics, Maria Sklodowska-Curie National Research Institute of Oncology, Roentgena 5, 02-781 Warsaw, Poland; 5 Institute of Biological Sciences, Cardinal Stefan Wyszynski University, 1/3 Kazimierza Wóycickiego St, 01-938 Warsaw, Poland

**Keywords:** Mycophenolic acid, Renal transplantation, VAMS, LC–MS/MS, Cross-validation, Clinical validation

## Abstract

**Background:**

Mycophenolic acid (MPA) is widely used in posttransplant pharmacotherapy for pediatric patients after renal transplantation. Volumetric absorptive microsampling (VAMS) is a recent approach for sample collection, particularly during therapeutic drug monitoring (TDM). The recommended matrix for MPA determination is plasma (PL), and conversion between capillary-blood VAMS samples and PL concentrations is required for the appropriate interpretation of the results.

**Methods:**

This study aimed to validate and develop a UHPLC-MS/MS method for MPA quantification in whole blood (WB), PL, and VAMS samples, with cross and clinical validation based on regression calculations. Methods were validated in the 0.10–15 µg/mL range for trough MPA concentration measurement according to the European Medicines Agency (EMA) guidelines. Fifty pediatric patients treated with MPA after renal transplantation were included in this study. PL and WB samples were obtained via venipuncture, whereas VAMS samples were collected after the fingerstick. The conversion from VAMS_MPA_ to PL_MPA_ concentration was performed using formulas based on hematocrit values and a regression model.

**Results:**

LC–MS/MS methods were successfully developed and validated according to EMA guidelines. The cross-correlation between the methods was evaluated using Passing-Bablok regression, Bland–Altman bias plots, and predictive performance calculations. Clinical validation of the developed method was successfully performed, and the formula based on regression was successfully validated for VAMS_MPA_ to PL_MPA_ concentration and confirmed on an independent group of samples.

**Conclusions:**

This study is the first development of a triple matrix-based LC–MS/MS method for MPA determination in the pediatric population after renal transplantation. For the first time, the developed methods were cross-validated with routinely used HPLC–DAD protocol.

**Graphical Abstract:**

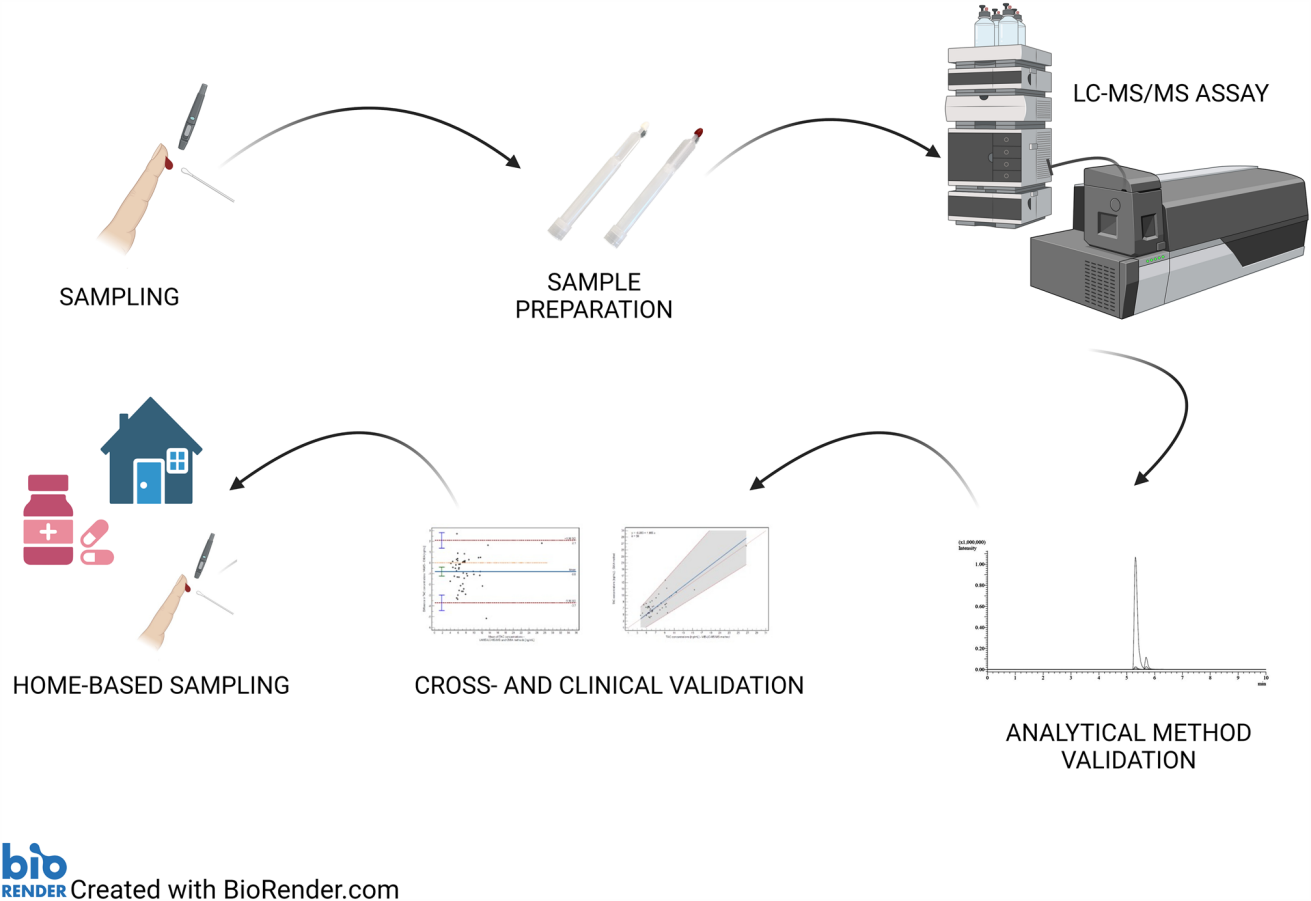

**Supplementary Information:**

The online version contains supplementary material available at 10.1007/s43440-023-00509-w.

## Introduction

From a long-term perspective, pediatric patients require optimal immunosuppressive therapy after kidney transplantation (KTx) to prevent toxicity and acute or chronic graft rejection [1, 2]. Pharmacotherapy is often based on tacrolimus (TAC), a cornerstone calcineurin inhibitor, combined with mycophenolate and corticosteroids. Mycophenolates are characterized by relatively high intra: and inter-individual variability [1, 2]. In such cases, therapeutic drug monitoring (TDM) of drug concentration in the blood is essential for dosage adjustment and individualized therapy. In the case of pediatric patients, MPA has registration for post-transplant treatment, with dosing 1200 or 900 mg/m^2^ of body surface area divided into two doses (concomitantly for cyclosporine and TAC, respectively). Some modifications may be made early after transplantation [1–3].

After administration, mycophenolate mofetil (MMF) is rapidly hydrolyzed to its active metabolite MPA. At the molecular level, MPA is a selective, specific, non-competitive, and reversible inhibitor of inosine monophosphate dehydrogenase (IMPDH) [1, 4]. In adults, MMF is monitored (but not obligatory) by measuring mycophenolic acid (MPA) in plasma; however, this is not implemented to the same degree in children [4]. The FDCC (Fixed-Dose Concentration Controlled) clinical trial with 62 pediatric patients treated with MMF showed comparable pharmacokinetic (PK) and efficacy to adults; however, children younger than 6 years needed more attention owing to potential adverse effects/toxicities [1, 4–6].

According to the IATDMCT (International Association of Therapeutic Drug Monitoring and Clinical Toxicology) recommendations, EDTA (ethylenediamine tetraacetic acid) plasma (PL), heparinized plasma (HP), and serum (SR) are suitable matrices for MPA determination [7]. MPA concentration is determined in TDM laboratories using immunoassays (IAs) or chromatographic methods. High-performance liquid chromatography (HPLC) conducted with ultraviolet spectrophotometry (UV) or a fluorescence detector (FLD) is frequently used in TDM laboratories as a routine method [1, 7]. On the other hand, the most attractive liquid chromatography–tandem mass spectrometry (LC–MS/MS) is considered a method routinely used in 40% of TDM laboratories. This method is regarded as the gold standard bioanalytical method for MPA determination. Owing to the higher specificity, sensitivity, and robustness of this method, some laboratories have used it for total- and free-MPA analyses in a complicated biological matrix [7, 8]. Chromatographic methods provide good separation of MPA, AcMPAG (mycophenolic acid acyl-glucuronide), and MPAG (mycophenolic acid glucuronide), as well as in the case of metabolite dissociation in the mass detector source of MPA [1, 7]. In contrast, cross-reactivity with metabolites is characteristic of IAs. The most popular enzyme multiplied immunoassay technique (EMIT), cloned enzyme donor immunoassay (CEDIA), and particle-enhanced turbidimetric inhibition immunoassay (PETINIA) are used as IAs for MPA determination [7]. The estimated typical C_0_ (trough concentration) of MPA ranges from 1 to 4 µg/mL [1].

The importance of volumetric absorptive microsampling techniques (VAMS) in the TDM of immunosuppressants is increasing annually [9, 10]. It is an alternative to frequent venipuncture, especially in the pediatric population. A strong point of this method may be the lack of phlebotomist presence during sampling and minimized biohazard risk. The most popular is the Mitra™ device produced by Neoteryx (Torrance, CA, USA), with different tip volumes (10, 20, and 30 µL) [9]. Instead of the dried-blood-spot (DBS) technique, VAMS is independent of the hematocrit (HT) interference and volcano effects. More theoretical information and analytical considerations regarding VAMS have been described in previously published reviews [10, 11]. Because MPA is almost exclusively found in the plasma fraction, capillary blood may be problematic for quantifying this drug. However, some approaches may be introduced to convert MPA_VAMS_ and MPA_PL_ concentrations. The equation MPA_VAMS_ / [1- HCT level] = MPA_PL_ is frequently used for conversion but is only suitable for drugs primarily binding with PL proteins [12, 13]. Some studies included the fraction bound to plasma protein (FBPP) factor in the above formula [14]. Other protocols assume that the equation obtained after the regression calculation (Passing-Bablok or Deming method) might be used for that conversion. However, this still depends on a specific set of data, and additional confirmation of the regression formula should be performed on the independent group of samples [12].

This study aimed to develop a method for determining MPA levels in samples obtained using a Mitra™- VAMS device. Additionally, for appropriate cross-validation and confirmation of the utility of VAMS for MPA determination, PL–LC–MS/MS and WB–LC–MS–MS analytical methods were developed and validated. Two approaches for concentration conversion were used and compared: the HT-dependent formula and the equation from the Passing-Bablok regression. The significance of the HT effect and the stability of the VAMS-loaded samples were also evaluated. Finally, successful cross and clinical validations were performed, and conversion between MPA concentrations obtained from capillary blood collected using VAMS was initially successful for the first time.

## Materials and methods

### Reagents and materials

The Supplementary File (Sect. 1) provides details regarding the reagents and materials used in this study.

### Equipment and liquid chromatography-tandem mass spectrometry conditions

The analytical platform used for MPA determination in VAMS, WB, and plasma consisted of an 8050 triple quadrupole mass detector (Shimadzu, Tokyo, Japan) coupled with a Nexera X2 UHPLC system (Shimadzu, Tokyo, Japan). Additionally, the supporting auxiliary equipment was adapted. The controller (CBM-20A), pump for isocratic and low-pressure gradient flow with a modified mixer (30AD), degasser unit (DGU-20A5R), autosampler with vial racks and cooling option (SIL-30AC), and thermostatic prominence column oven (CTO-20AC) were used. The chromatographic column (150 × 3.00 mm, 2.70 μm) Poroshell 120-EC-C_18_ and complementary guard pre-column named Poroshell UHPLC EC-C_18_ (4.60 × 3.00 mm, 2.70 μm) were delivered by Agilent (Santa Clara, CA, USA). For appropriate chromatographic separation, the above column was maintained at 40 °C. The final mobile phase was a gradient mixture of two solutions: A (pure deionized water) and B (methanol), each with 2.5 mM ammonium fluoride and 0.05% formic acid addition. The summary flow rate was set at 0.50 mL/min during the following 10-min LC gradient program:

1. From injection to 1.49 min—a total volume of 95% phase A and 5% phase B;
2. Between 1.50 and 7.49 min—the total volume is 100% of phase B;
3. Between 7.49 min and the end of the run—equilibration with the same as at the initial point.

The autosampler cooler was set at 5 ℃. The injection volume during assays was set as 10 µL with a 5.00 μL/sec speed of delivery into the MS detector for each examined matrix.

Optimization of source parameters and multiple pair monitoring (MRM) was established with LabSolution 5.97 (Shimadzu, Kyoto, Japan) method creator or experimentally according to peak parameter observation. The MRM pairs (target-quantitative and control-qualitative pairs) are described in Table 1, along with the retention time of the analytes (RT). The proton adduct [M + H]^+^ was used for MPA and MPA-d_3_ monitoring, whereas [M + NH_4_]^+^ was used for MPAG qualitative determination, both in positive-ion mode with electrospray ionization (ESI +). The single-event time was set to 0.062 ms.

**Table 1:** Multiple pair monitoring mass spectrometry parameters with retention times of analytes

Analyte	Precursor ion (*m*/*z*)	Product ion (*m*/*z*)	Collision energy (eV)	Dwell time (msec)	Retention time (min)
MPA	321.10 321.10	275.00 285.00	− 15 − 20	28 28	5.58
MPA-d_3(internal standard)_	324.00 341.00	210.00 210.00	− 22 − 35	28 28	5.58
MPAG^(only monitored)^	514.21 514.21	207.04 303.13	− 38 − 18	28 28	5.20

The first line in the table for each analyte describes the parameters for the quantitative pair, while the second describes the parameters for the qualitative pair. MPA-d_3_ was used as an internal standard in MPA quantification using LC–MS/MS method. MPAG was only monitored to avoid in-source fragmentation to free MPA

*MPA* mycophenolic acid, *MPA-d*_*3*_ deuterated mycophenolic acid, *MPAG* mycophenolic acid glucuronide

The first MRM pair was used for validation and patient sample calculations (excluding MPAG, which was only monitored), whereas the second pair was set as the control pair.

The electrospray voltage of the detector was set to 3 kV, and the interface, desolvation line (DL), and heat block (HB) temperatures were set to 150, 250, and 400 ℃, respectively. Argon was used as the collision-induced dissociation gas at 270 kPa pressure. Nitrogen was used as the drying gas and nebulizing gas (flow rates: 5 and 3 L/min, respectively), whereas air was used as the heating gas (flow rate: 14 L/min).

### Calibrators and quality control preparation

The details of the calibrators and quality controls (QC) preparation are provided in the Supplementary File (Sect. 2).

### Patients and sampling protocol

Fifty pediatric renal transplant recipients participated in this study. The children were treated at the Kidney Transplantation Outpatient Clinic, Children’s Memorial Health Institute (CMHI), Warsaw. The patients were treated with an immunosuppressive regimen of TAC (Advagraf^®^ or Prograf^®^, Astellas, Warsaw, Poland), MMF (CellCept^®^, Roche AG, Basel, Switzerland), and glucocorticosteroid drugs. WB-anticoagulant with EDTA was obtained before the next daily dose of immunosuppressive agents (MPA and TAC) via classic venous collection (part of the sample was used for PL collection) during regular post-transplant follow-up visits between November 2022 and February 2023. Samples of WB and PL were stored at – 20 ℃ pending the LC–MS/MS assay. Simultaneously, VAMS samples (finger puncture by a lancet) were collected by a Medical Doctor or Nurse and analyzed during the next two weeks (held at RT in the dark; stability was confirmed in the presented study (“Validation of methods for MPA determination in whole blood, plasma, and volumetric-absorptive microsampling samples”). The pediatric participants and their parents or legal guardians provided informed consent before sampling. This study was conducted in accordance with the Declaration of Helsinki, Council for International Organizations of Medical Sciences Guidelines, and Good Clinical Practice (GCP). This study was approved by the Bioethics Committee of the Children’s Memorial Health Institute in Warsaw (approval number and date: 36/KBE/2022, 19 October 2022).

### Sample preparation

The details of the sample preparation are provided in the Supplementary File (Sect. 3).

### Method validation

The validation process was performed according to the European Medicines Agency guidelines for bioanalytical method validation published in January 2023 and the International Association of Therapeutic Monitoring and Clinical Toxicology (IATDMCT) guidelines for DBS-method development and consensus regarding mycophenolate treatment [1, 13, 16]. For each validated method (WB, PL, and VAMS), the following parameters were verified: selectivity, lower limit of quantification (LLOQ), calibration and linearity, accuracy and precision, carry-over, matrix effect with process efficiency and recovery, autosampler stability, long-term stability of the VAMS samplers, and short- and long-term stability of the final sample and working solutions. HCT-independence experiments for MPA in VAMS and the incurred sample reanalysis (ISR) protocol were also performed. Passing-Bablok regression, Bland–Altman plots, predictive bias, and other correlation parameters were evaluated for clinical validation.

Blank samples (without analytes) were analyzed from six different donors for each tested matrix (WB, PL, and VAMS) for selectivity evaluation. Adequate selectivity was achieved when the response of the interfering components was lower than 20% for the analyte (LLOQ) and 5% for the IS [16].

Linearity was calculated for each calibration method using linear 1/*x* weighting in the 0.10–15.0 µg/mL range based on the following concentration points: 0.10, 0.50, 1.0, 2.5, 5.0, 10.0, 15.0 µg/mL. Calibration curves were constructed for calibration and linearity parameter evaluation. Blank and zero samples were simultaneously prepared during calibration set determination. Blank samples (without IS and MPA) and zero samples (without MPA, but with IS) were prepared for interference determination during the chromatographic run.

The carry-over effect was experimentally evaluated in 20 different runs by directly injecting the HQC sample immediately before the blank sample without the analytes. According to the EMA validation guidelines, the acceptance criteria for MPA are less than 20% and less than 5% for IS [16].

Accuracy is the percentage ratio determined to the reference concentration values, and precision is the percentage coefficient of variation (CV%). This study evaluated parameters within and between runs (intra- and inter-day) in six repetitions for each analyzed matrix. Following EMA guidelines, the acceptance criteria for both parameters require that the mean is within 15% of the reference value, whereas the LLOQ should be within 20% [16].

The stability parameter was evaluated for samples in the autosampler (1, 3, and 5 days after storage at 5 ℃) and in the short term during preparation (pre- and post-preparation experiments for LQC and HQC). Additionally, the influence of conditions during the long period of loaded VAMS samplers was examined for LQC and HQC (1, 2, 4, 6, and 8 weeks, at RT and 4 °C). The long-term stability was assessed only for the working solutions, and the samples were not tested according to the TDM specifications after preparation. Based on stability, all procedures were repeated six times for each MPA determination method. The stability may be acceptable if the differences are within the ± 15% nominal value range [13, 16].

Based on the formulas presented by Taylor et al. and Zhou et al., the matrix effect (ME) was calculated after post-extraction addition experiment testing for six different sources (separately for WB, PL, and VAMS samples, in LQC and HQC levels). Similarly, the process efficacy (PE) and absolute recovery (AR) ratios (expressed as percentages) were evaluated for MPA and IS, including each matrix [15, 17, 18].

According to the comedication, sample inhomogeneity, and metabolite presence, an Incurred Sample Reanalysis (ISR) experiment should be performed for potential differences in observations between calibrators and samples. This experiment used patient samples from separate analytical runs performed on different days. This was repeated twice for each sample [*n* = 10] using the VAMS-LC–MS/MS, PL-LC–MS/MS, and WB-LC–MS/MS methods. The percent difference between the initial concentration value and the reanalysis concentration level should be at most 20% of the mean for at least 67% of repetitions. The differences were calculated using the following formula: (repeat value–initial value)/mean value of both measurements and expressed as a percentage ratio [15, 16].

The hematocrit effect (HE) was evaluated as in our previous TAC study [15]. Thus, assuming that HE caused differences in MPA concentrations observed between WB and VAMS samples, the correlation for differences in HE values was evaluated mathematically.

**Fig. 1:**
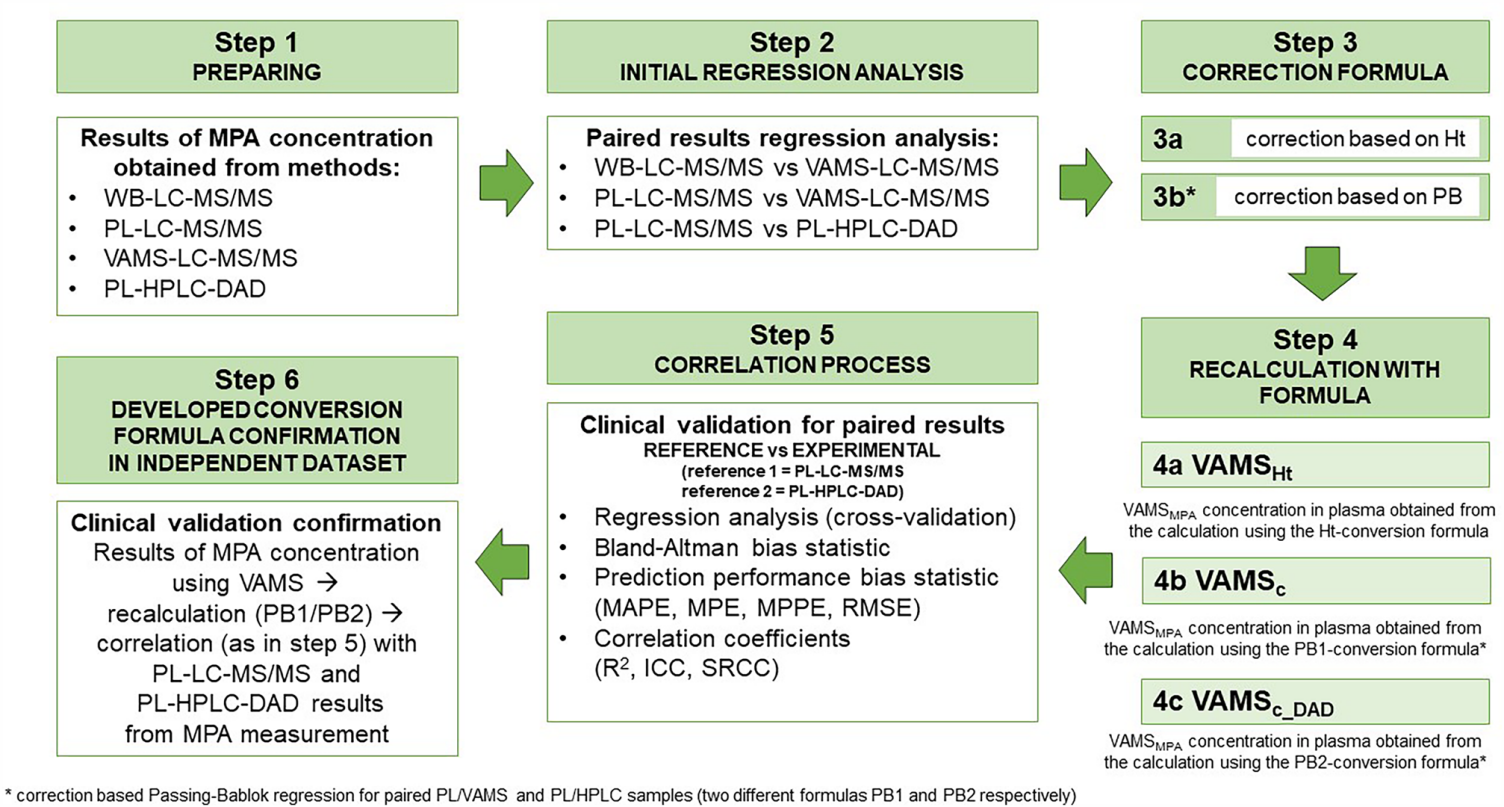
Phases of cross and clinical validation are presented as the flowchart with the procedure that should be followed for each of the compared methods and paired samples. The algorithm is presented by the example of the data presented study. *WB* whole blood, *PL* plasma, *VAMS* volumetric absorptive microsampling, *LC–MS/MS* liquid chromatography-tandem mass spectrometry, *HPLC–DAD* high-performance liquid chromatography with diode array detection, *PB* Passing-Bablok regression, *Vams*_*hi*_ mycophenolic acid concentration corrected with hematocrit, *VAMS*_*c*_ mycophenolic acid concentration corrected with regression, *VAMS*_*c_DAD*_ mycophenolic acid concentration corrected with regression, *MPE* median prediction error, *MPPE* median percentage prediction error, *RMSE* root mean squared prediction error, *MAPE* median absolute percentage prediction error, *R*^2^ Pearson’s correlation, *SRCC* Spearman rank correlation coefficient, *ICC* Intraclass correlation coefficient

MPA concentration measurements (Fig. 1, step 1) were analyzed by cross-validation. Paired methods were evaluated using statistical tools (according to EMA guidelines) using Passing-Bablok regression, Bland–Altman plots, correlation, and predictive performance methods. Differences between the methods were assessed using the Passing-Bablok regression. Two methods were set as equivalent, while 1 was within the 95% CI range for slope and 0 was within the 95% CI range for intercept [19]. Based on EMA guidelines, differences between methods checked with the Bland–Altman plot should be lower than 20% for 67% of the analyzed pairs [16, 20]. Instead, the clinical acceptance criterion for paired sample bias was < 15% for 67% of the analyzed pairs [13]. Correlation between the methods was assessed using Pearson’s and intraclass correlation coefficients (ICC). Following the IATDMCT guidelines for predictive performance, the median prediction error (MPE), median percentage prediction error (MPPE), root mean squared prediction error (RMSE), and median absolute percentage prediction error (MAPE) were calculated (the predictive parameters should be lower than 15%) [13, 21]. The above analysis of the results is shown in steps 2 and 3 in Fig. 1.

The results obtained were used in the clinical validation process based on the guidelines of Capiau et al. for DBS validation (Fig. 3, steps 4–6) [13]. MPA concentrations were recalculated using the formula obtained during the cross-validation. The results were analyzed again using the same statistical tools, similar to the cross-validation process.

An independent set of data—the results of MPA determination obtained using prescribed analytical methods—was used for the regression formula validation using the above-mentioned statistical methods.

During the chromatogram analysis and evaluation, LabSolutions software (version 5.97, Shimadzu, Tokyo, Japan) was used for peak marking and calibration curves by linear 1/x weighting with the correlation coefficient of the calibration curve (*R*^2^) calculation. MedCalc (version 20.218, MedCalc, Ostend, Belgium) and GraphPad Prism (9.5.1, Boston, MA, USA) were used for graph preparation, cross-validation, and clinical comparison. All data presented in this study are expressed as the arithmetic mean ± standard deviation (SD) or the coefficient of variation (CV).

### Routinely used high-performance liquid chromatography coupled with diode array detection method (HPLC–DAD) description

The HPLC method using DAD detection was routinely performed in the CMHI for MPA concentration determination in the patients treated with MMF. After MPA extraction from the serum or plasma using the liquid–liquid extraction (LLE) method with methylene chloride and butanol (98:2, v/v) evaporation using nitrogen, the dry residue was dissolved in the mobile phase, and 50 µL of the sample was injected into a C_18_ column with UV detection at 214 nm. A mixture of acetonitrile and water (36%) was used as the mobile phase at a 1.6 mL/min flow rate; retention time values were 5 and 7 min (for MPA and IS, respectively), and a total run time of 10 min. Oxazepam was used as the structurally unrelated internal standard (URIS). The results were calculated using the F-factor, set at 2.6 (as the MPA-to-IS peak area ratio). The Pharmacokinetics Laboratory of CMHI regularly participated in the LGC Group (Laboratory of the Government Chemist, Teddington, UK) immunosuppressant proficiency scheme (the therapeutic reference range was successfully calibrated in each round and set at 1.0–3.5 µg/mL).

## Results

### Method development

Under the established chromatographic conditions, the run time was set as 10 min, and the retention time values for the analytes were determined (see chromatograms in Supplementary File–Sect. 4). MPAG was used only for co-elution and MS source-detector optimization in the presented experiments. According to the potential MPAG in-source dissociation to MPA, the MS working conditions were set experimentally based on the ratio between the MPAG and MPA peaks and the in-source product of the dissociation peak [22]. Additionally, MPAG and MPA were separated chromatographically. Different solutions were tested for extraction optimization: pure methanol or acetonitrile and their mixtures with water and purified water. According to our previous study, pure water is the best solvent for MPA extraction from VAMS. Based on our experience, the precipitation mixture is optimal for all analyte extractions from the VAMS tip [15]. The drying time was optimal during the 1 h cycle. LC–MS/MS methods for all matrices were established using the chromatographic and apparatus conditions described above. As organic mobile phases, mixtures of acetonitrile and methanol (50:50) and pure methanol, with formic acid and ammonium formate or ammonium fluoride addition, were tested. The optimal mobile phase was methanol with formic acid and ammonium fluoride addition, based on the experiments. Ammonium fluoride was added to the mobile phase for appropriate monitoring of MPAG according to the peak height, which was experimentally optimised. For chromatographic separation, different columns were tested: Zorbax Eclipse Plus-C_18_ (2.1 × 100 mm, 1.8 µm; Agilent, Santa Clara, CA, USA), Kinetex-C_18_ (4.6 × 100 mm, 2.6 µm; Phenomenex, Torrance, CA, USA), Hypurity-C_18_ (50 × 2.10 mm, 3 µm; ThermoScientific, Waltham, MA, USA) and mentioned above—Poroshell. Only the latter method ensured appropriate separation and optimal retention times.

Representative chromatograms of the blank sample, HQC, and LQC calibrators are presented in the Supplementary File (Sect. 4).

### Validation of methods for MPA determination in whole blood, plasma, and volumetric-absorptive microsampling samples

The LC–MS/MS methods for WB and PL were validated in the 0.10–15 µg/mL calibration range, using MPA-d_3_ as the IS.

#### Whole blood and plasma

In both matrices, linearity was assessed based on ten calibration curves using linear 1/*x* weighting. The summarized mean *R*^2^ was 0.9984 ± 0.0013, and the mean calibration equation was *y* = 0.9821x + 0.011 for WB and 0.9994 ± 0.0013 and *y* = 1.004*x* – 0.037 for PL. A calibration curve was constructed using seven concentration levels, with zero and blank samples. Signal-to-noise ratio (S/N) was used for LLOQ experimentally establishment–0.10 µg/mL in WB and PL. Chromatograms were analyzed according to interferences caused by unknown or endogenous substances from the matrix (lower than 15% of 0.10 µg/mL in both methods). The precision and accuracy of MPA concentrations for both matrices at the LQC, MQC, and HQC were within the EMA and IATDMCT acceptance ranges (accuracy within 85–115%, imprecision less than 15%, and less than 20% for LQC, and CV < 10% respectively) [7, 13, 16]. The carry-over effect was insignificant and fulfilled the EMA criteria for MPA and IS (0.9838 ± 0.4157% and 0.223 ± 0.082%, respectively) [15]. Similarly, the plasma carry-over effect was insignificant for MPA and IS (0.823 ± 0.452% and 0.268 ± 0.018%, respectively). WB and PL samples were stable at 5 °C for LQC and HQC in the autosampler (*n* = 6; initial day 1–3 and after the 5-day checkpoint), fulfilled the acceptance criteria, were satisfactory, and were stable before and after extraction [*n* = 6; for LQC and HQC]. The ME, AR, and PE were calculated after the pre- and post-extraction experiments. The above parameters characterized the matrix effects in the WB, and the PL fulfilled the EMA acceptance criteria [16]. For the ISR experiment, the %-difference between pairs of the sample was lower than 20% for 80% of WB samples (mean: − 4.07 ± 4.85) and 70% of PL samples (mean: − 6.68 ± 7.15). The summarized validation results are presented in the Supplementary File (Sect. 5).

#### Volumetric-absorptive microsampling samples (VAMS)

Linearity was assessed based on 10 calibration curves (each with seven concentration levels, with zero and blank samples) using linear 1/*x* weighting; the summarized mean *R*^2^ was 0.998 ± 0.001, and the calibration equation was *y* = 0.9678*x* – 0.005. In this case, signal-to-noise ratio (S/N) was also used for LLOQ experimentally establishment–0.10 µg/mL. Unknown or endogenous substances did not interfere in assays (lower than 15% of 0.10 µg/mL). The precision and accuracy evaluation results of MPA concentrations at the LQC, MQC, and HQC were within the EMA and IATDMCT acceptance ranges (accuracy within 85–115%, imprecision less than 15% and less than 20% for LQC, and CV < 10% respectively) [7, 13, 16]. The carry-over effect was insignificant and fulfilled the EMA criteria for MPA and IS (0.5070 ± 0.1236% and 0.1600 ± 0.041%, respectively) [15]. Stability at 5 °C for LQC and HQC in the autosampler (*n* = 6; initial day 1–3 and after 5-day checkpoint) fulfilled the acceptance criteria and was satisfactory, as well as stability before and after extraction [*n* = 6; for LQC and HQC]. The ME, AR, and PE were calculated after the pre- and post-extraction experiments. The parameters characterized by matrix effects fulfilled the EMA acceptance criteria [15]. The summarized validation results are presented in the Supplementary File (Sect. 5).

**Fig. 2:**
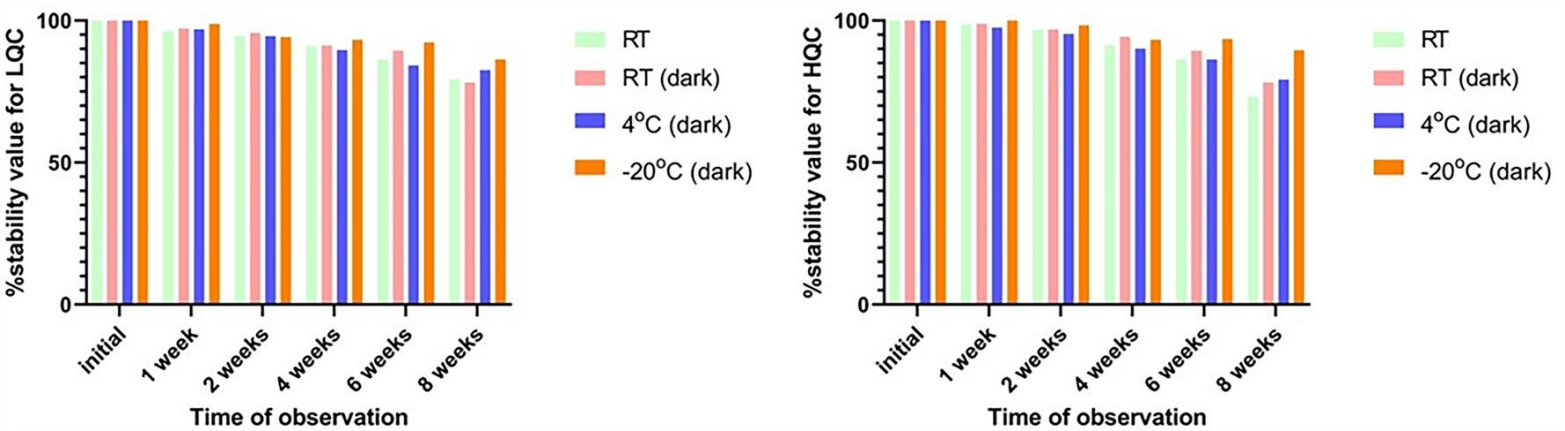
Evaluation of VAMS stability under different conditions [*n* = 4]. The stability was evaluated using two calibration levels of mycophenolic acid—LQC and HQC (0.35 µg/mL and 12.50 µg/mL, respectively) in different storage conditions (at RT with access to light and in the dark, and at 4 ℃ and – 20 ℃, both in the dark). The analyte in the VAMS sampler was stable during the six weeks in all storage places at both calibration levels. Stability is expressed as a percentage ratio to initial stability (100%). *RT* room temperature, *RT* (dark) room temperature in the dark condition, *LQC* lower quality control concentration, *HQC* higher quality control concentration, *VAMS* volumetric absorptive microsampling

Loaded VAMS sampler stability was evaluated for LQC and HQC under different storage conditions (at RT in the dark, at RT with access to light, and at 4 ℃ and – 20 ℃, both at night) with a variable time of observation (1, 2, 4, 6, and 8 weeks). The analyte was stable at both concentrations during six weeks of storage at RT and 4 ℃ (both in the dark). Only in the case of freezing (− 20 ℃) VAMS was stable during the observation period (8 weeks). The results of the stability tests are shown in Fig. 2.

**Fig. 3:**
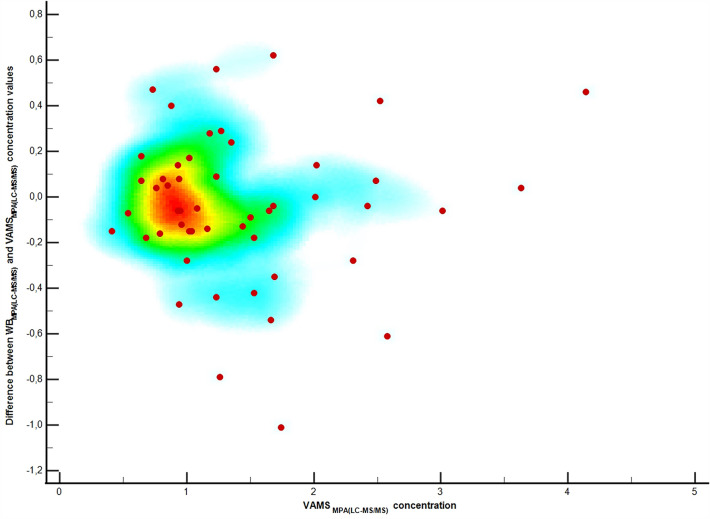
Scatter diagram showing the effect of the hematocrit (presented as cold/hot map of correlation between WB_MPA(LC–MS/MS)_ and VAMS_MPA(LC–MS/MS)_ concentrations differences). The correlation coefficient of the presented data was lower than zero (− 0.02596, − 0.31717 to 02696 for 95% CI), confirming that HT level does not influence analyte recovery from the VAMS sampler. *WB*_*MPA(LC–MS/MS)*_ MPA concentration in whole blood measured using tandem-mass liquid-chromatography analytical method (without correction), *VAMS *_*MPA(LC–MS/MS)*_ MPA concentration in volumetric absorptive micro sample measured using tandem-mass liquid-chromatography analytical method (without correction), *HT* hematocrit level, *VAMS* volumetric absorptive microsampling

The effect of HT was evaluated for each patient included in the study. The Pearson correlation coefficient was − 0.02596 (− 0.3171 to 0.2696, 95% CI), confirming that no correlation was observed in this case. The results are presented as a scatter diagram in Fig. 3.

For the ISR experiment, the % difference between pairs of samples was lower than 20% for 80% of samples (mean: − 10.73 ± 6.86) and consequently fulfilled EMA acceptance criteria [13, 16].

### Patient’s clinical data

An overview of the patient’s demographic data is presented in the Supplementary File (Sect. 6).

### Clinical and cross-validation

**Fig. 4:**
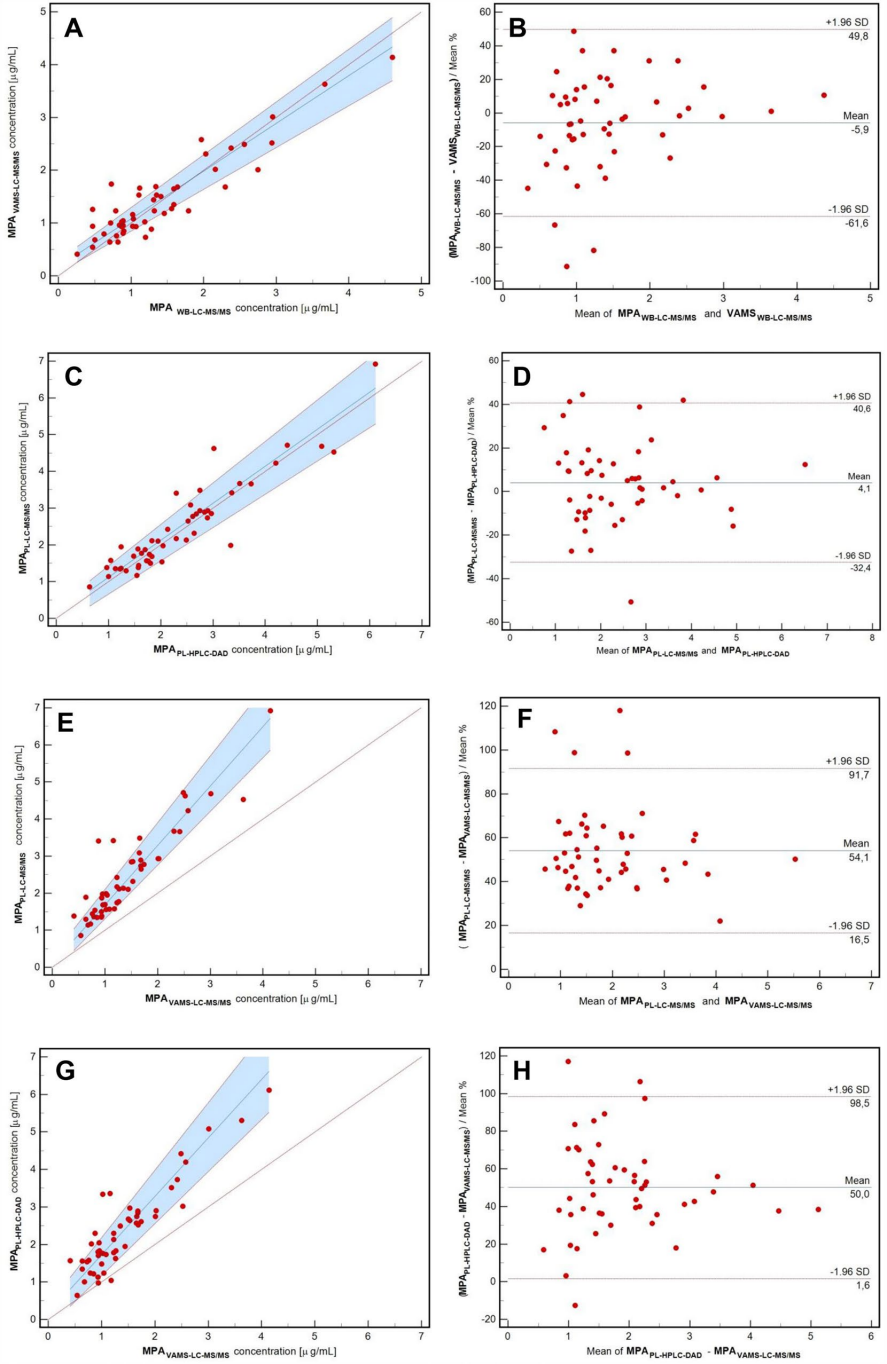
Passing-Bablok regression curves (**a, c, e, g**) and Bland–Altman plots (**b, d, f, h**) for cross-validation of the methods before conversion using hematocrit and regression formulas. Compared methods are described on the axis in each presented case. Data are presented as red circle points for all paired samples. The regression line, diagonal line, and confidence interval curve are presented as blue, red dotted, and blue area, respectively, on Passing-Bablok regression curves (**a, c, e, g**). The limit of agreement and the average difference are presented in red dotted lines and blue line, respectively, on Bland–Altman plots (**b, d, f, h**). In contrast, average bias is presented as a percent value with ± 1.96SD range. *VAMS* volumetric absorptive microsampling, *LC–MS/MS* liquid chromatography-tandem mass spectrometry, *HPLC–DAD* high-performance liquid chromatography with diode array detection, *MPA*_*VAMS-LC–MS/MS*_ mycophenolic acid concentration in VAMS sample determined LC–MS/MS, *MPA*_*WB-LC–MS/MS*_ mycophenolic acid concentration in whole blood sample determined LC–MS/MS, *MPA*_*PL-LC–MS/MS*_ mycophenolic acid concentration in plasma sample determined LC–MS/MS, *MPA*_*PL-HPLC–DAD*_ mycophenolic acid concentration in plasma sample determined HPLC–DAD

The quality of the VAMS samples was satisfactory (only two tips were excluded from the study because of the unloading of capillary blood). Clinical and cross-validation were based on 150 samples (three samples from each patient included in the study: WB, PL, and VAMS concentrations). A summary of the cross-validation process is provided in Fig. 4 as regression and Bland–Altman graphs. The data from the statistical analysis of the paired compared methods are shown in Table 2.

**Table 2:** Cross-validation and clinical validation results—before and after conversion using recalculating formulas (based on: individual hematocrit level and validated Passing-Bablok regression model)

Statistic	Before conversion (cross-validation)				After conversion (clinical validation)		
	MPA_VAMS-LC–MS/MS_ *vs* MPA_WB-LC–MS/MS_	MPA_VAMS-LC–MS/MS_ *vs* MPA_PL-LC–MS/MS_	MPA_PL-HPLC–DAD_ *vs* MPA_PL-LC–MS/MS_	MPA_VAMS-LC–MS/MS_ *vs* MPA_PL-HPLC–DAD_	MPA_VAMSHt-LC–MS/MS_ *vs* MPA_PL-LC–MS/MS_	MPA_VAMSc-LC–MS/MS_ *vs* MPA_PL-LC–MS/MS_	MPA_VAMSc_DAD-LC–MS/MS_ *vs* MPA_PL-HPLC–DAD_
Passing–Bablok analysis (regression formula)							
Passing-Bablok-regression formula	C_VAMS_ = 0.90·C_WB_ + 0.17	C_PL_ = 1.60·C_VAMS_ + 0.08	C_HPLC_ = 1.01·C_PL_ + 0.11	C_HPLC_ = 1.56·C_PL_ + 0.17	C_PL_ = C_VAMS**Ht**_ — 0.13	C_PL_ = C_VAMS**c**_ — 0.01	C_VAMS**c_DAD**_ = 1.01·C_HPLC_ —0.02
Intercept (A)	0.17 (− 0.06 to 0.29)	0.08 (− 0.14 to 0.29)	0.11 (− 0.24 to 0.30)	0.17 (− 0.22 to 0.38)	− 0.13 (− 0.35 to 0.03)	− 0.01 (− 0.27 to 0.19)	− 0.03 (− 0.35 to 0.31)
Slope (B)	0.90 (0.79–1.00)	1.60 (1.44–1.80)	1.01 (0.91–1.13)	1.56 (1.38–1.78)	1.00 (0.92–1.09)	1.00 (0.90–1.11)	1.01 (0.89–1.15)
Bland–Altman bias analysis (based on plot and %-differences bias)							
%-mean difference bias (− 1.96SD; + 1.96SD)	− 6.80% (− 61.50 to 47.90%)	54.07% (48.62–59.53%)	+ 4.08% (− 1.21 to 9.37%)	50.00% (1.60–98.50%)	+ 10.90% (5.24–16.56%)	4.76% (− 1.11 to 10.64%)	− 1.01% (− 8.26 to 6.24%)
% of paired samples fulfilled the EMA criteria (< 20%)	70%	0%	12%	12%	88%	86%	72%
% of paired samples fulfilled the IATDMCT criteria (< 15%)	60%	0%	6%	4%	78%	72%	67%
Predictive analysis performance factors							
MPE	− 0.05	− 0.92	− 0.12	− 0.81	− 0.12	− 0.01	0.00
MPPE [%]	− 5.50	85.00	1.90	67.54	7.03	9.20	2.60
RMSE [%]	15.00	92.00	12.00	81.54	12.14	12.21	13.00
MAPE [%]	13.69	66.65	8.25	62.42	14.02	13.38	20.24
Correlation coefficients							
Pearson’s (*R*^2^)	0.92 (0.87–0.96; 95% CI)	0.92 (0.86–0.95; 95% CI)	0.93 (0.88–0.96; 95% CI)	0.90 (0.84–0.95; 95% CI)	0.95 (0.85–0.97; 95% CI)	0.92 (0.87–0.96; 95% CI)	0.91 (0.85–0.95; 95% CI)
Spearman (SRCC)	0.81 (0.68–0.89; 95% CI)	0.88 (0.80–0.93; 95% CI)	0.92 (0.86–0.95; 95% CI)	0.83 (0.73–0.90; 95% CI)	0.91 (0.84–0.95; 95% CI)	0.88 (0.80–0.93; 95% CI)	0.84 (0.73–0.90; 95% CI)
Intraclass (ICC)	0.91 (0.85–0.95; 95% CI)	0.84 (0.73–0.90; 95% CI)	0.96 (0.93–0.98; 95% CI)	0.83 (0.74–0.91; 95% CI)	0.94 (0.90–0.97; 95% CI)	0.92 (0.87–0.95; 95% CI)	0.91 (0.85–0.95; 95% CI)

Data are presented as mean with range and confidence interval (95% CI)

*VAMS* volumetric absorptive microsampling, *LC–MS/MS* liquid chromatography-tandem mass spectrometry, *HPLC–DAD* high-performance liquid chromatography with diode array detection, *MPA*_*VAMS-LC–MS/MS*_ mycophenolic acid concentration in VAMS sample determined LC–MS/MS, *MPA*_*WB-LC–MS/MS*_ mycophenolic acid concentration in whole blood sample determined LC–MS/MS, *MPA*_*PL-LC–MS/MS*_ mycophenolic acid concentration in plasma sample determined LC–MS/MS, *MPA*_*PL-HPLC–DAD*_ mycophenolic acid concentration in plasma sample determined HPLC–DAD, *MPA*_*VAMSHt-LC–MS/MS*_ mycophenolic acid concentration corrected with hematocrit, *MPA*_*VAMSc-LC–MS/MS*_ mycophenolic acid concentration corrected with regression, *MPA*_*VAMSc_DAD-LC–MS/MS*_ mycophenolic acid concentration corrected with regression, *IATDMCT* International Association of Therapeutic Drug Monitoring and Clinical Toxicology, *EMA* European Medicines Agency, *MPE* median prediction error, *MPPE* median percentage prediction error, *RMSE* root mean squared prediction error, *MAPE* median absolute percentage prediction error, *R*^2^ Pearson’s correlation, *SRCC* Spearman rank correlation coefficient, *ICC* Intraclass correlation coefficient

Passing-Bablok regression demonstrated equivalence between VAMS-LC–MS/MS and WB-LC–MS/MS (Fig. 4a and Table 2). The Bland–Altman plot describes the mean bias for the paired methods shown in Fig. 4b. The mean difference in the case of WB/VAMS revealed that the methods were acceptable according to EMA acceptance criteria (< 20%) but not in the case of IATDMCT criteria (< 15%). The WB method for MPA determination has been validated to check the equivalence between concentrations in WB and VAMS samples [7, 13, 16]. Parameters characterized by predictive performance fulfilled the IATDMCT criteria in the case of VAMS-LC–MS/MS and WB-LC–MS/MS method confirmation; the values were lower than 15%.

Interchangeability has also been confirmed for paired results from routinely used HPLC–DAD and reference LC–MS/MS methods (regression: *y* = 1.01*x* + 0.11; slope: 1.01, 0.91 to 1.13; intercept: 0.11, − 0.24 to 0.30, 95% CI; see Fig. 4c). The mean bias based on the Bland–Altman plot fulfilled the EMA and IATDMCT criteria (Fig. 4d) [12, 15]. High correlation factors were also observed: 0.93 and 0.96 (Pearson and ICC). The predictive performance ratio was < 15%.

To establish the correction formula, the VAMS/PL MPA concentration was recalculated according to two approaches: regression Eq. (1) or formula with HT (2) [13]:

1 $${\text{The correction formula}}: \, y \, = { 1}.{6}0x \, + \, 0.0{8 }\left( {{\text{where }}y \, = {\text{ C}}_{{{\text{MPA}}}} {\text{in PL and }}x \, = {\text{ C}}_{{{\text{MPA}}}} {\text{in VAMS}}} \right){\text{ is presented in Fig}}.{\text{ 4e}}$$

**Fig. 5:**
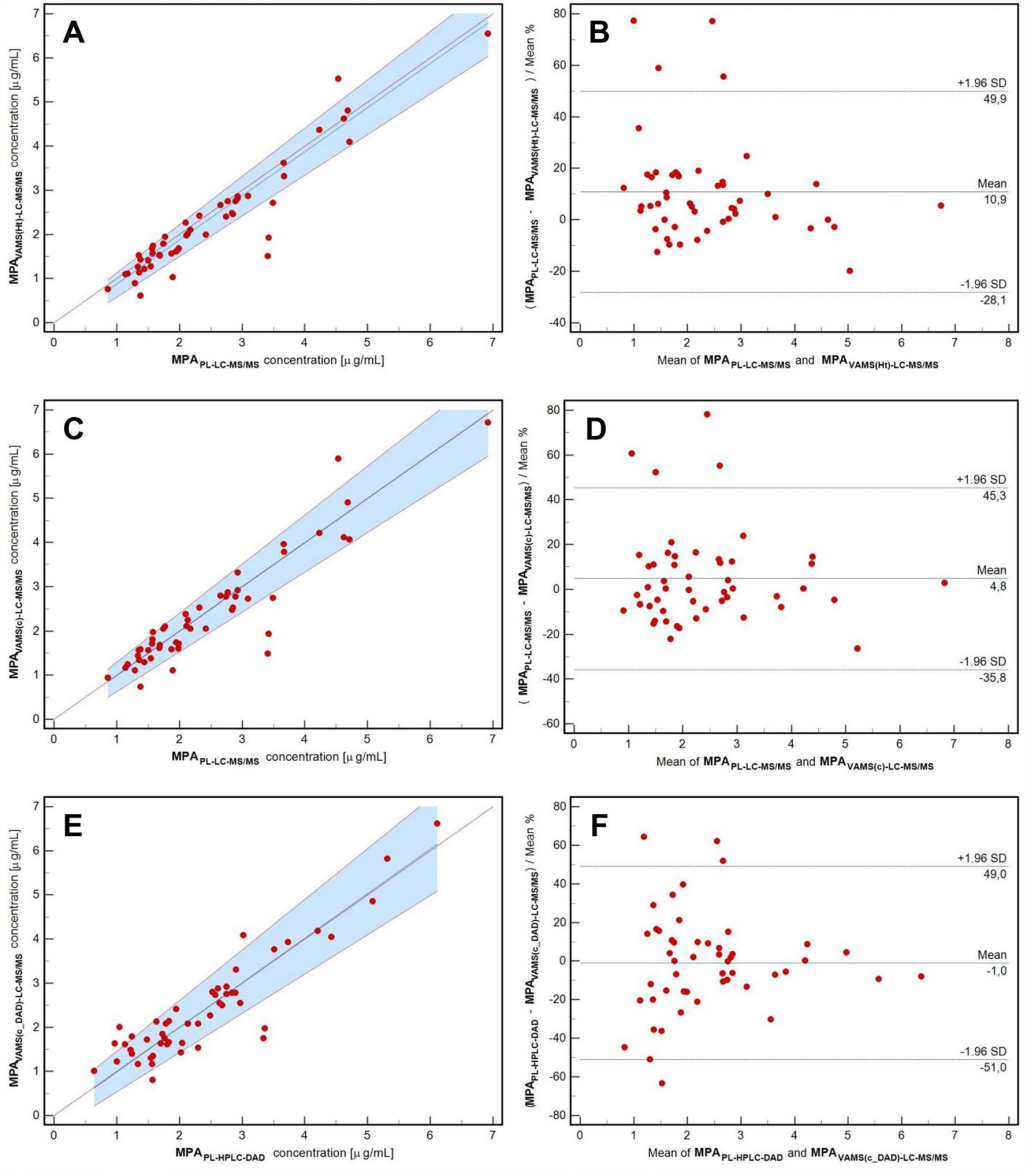
Passing-Bablok regression curves (**a, c, e**) and Bland–Altman plots (**b, d, f**) for clinical validation of the methods after conversion before conversion using hematocrit and regression formulas. Compared methods are described on the axis in each presented case. Data are presented as red circle points for all paired samples. The regression line, diagonal line, and confidence interval curve are presented as blue, red dotted, and blue area, respectively, on Passing-Bablok regression curves (**a, c, e**). The limit of agreement and the average difference are presented in red dotted lines and blue line, respectively, on Bland–Altman plots (**b, d, f**). In contrast, average bias is presented as a percent value with ± 1.96SD range. *VAMS* volumetric absorptive microsampling, *LC–MS/MS* liquid chromatography-tandem mass spectrometry, *HPLC–DAD* high-performance liquid chromatography with diode array detection, *MPA*_*VAMS-LC–MS/MS*_ mycophenolic acid concentration in VAMS sample determined LC–MS/MS, *MPA*_*WB-LC–MS/MS*_ mycophenolic acid concentration in whole blood sample determined LC–MS/MS, *MPA*_*PL-LC–MS/MS*_ mycophenolic acid concentration in plasma sample determined LC–MS/MS, *MPA*_*PL-HPLC–DAD*_ mycophenolic acid concentration in plasma sample determined HPLC–DAD, *MPA*_*VAMSHt-LC–MS/MS*_ mycophenolic acid concentration corrected with hematocrit, *MPA*_*VAMSc-LC–MS/MS*_ mycophenolic acid concentration corrected with regression, *MPA*_*VAMSc_DAD-LC–MS/MS*_ mycophenolic acid concentration corrected with regression

Regression equation (corrected MPA_VAMSc_/MPA_PLASMA_): *y* = *x* – 0.01 (slope: 1.00, 0.90, 1.11, 95% CI; intercept: − 0.01, − 0.27 to 0.19, 95% CI), is presented in Fig. 5a.

Bland–Altman plot—mean bias: 4.76% (− 1.11 to 10.64, 95% CI) – 86% of paired samples fulfilled EMA criteria (< 20%), while 72% of paired samples fulfilled the IATDMCT criteria (< 15%), presented in Figures: 4f (before conversion) and 5b (after conversion).

Predictive performance: MPPE, MAPE, and RMSE were lower than 15% (mean values: 9.20%, 13.38%, and 12.21%, respectively). Paired results were highly correlated.

2 $${\text{Correction formula}}:{\text{ C}}_{{{\text{VAMS}} - {\text{MPA}}}} / \, \left[ {{1} - {\text{ HCT level}}} \right] \, = {\text{ C}}_{{{\text{PL}} - {\text{MPA}}}}$$

The regression equation (corrected C_MPA_Ht_/C_MPA_PLASMA_): *y* = *x* – 0.13 (slope: 1.00, 0.92–1.09, 95% CI; intercept: − 0.13, − 0.35 and 0.03, 95% CI) is presented in Fig. 5c.

Bland–Altman plot mean bias: + 10.90% (5.24 to 16.56, 95% CI) – 88% of paired samples fulfilled the EMA criteria (< 20%), whereas 78% of paired samples fulfilled the IATDMCT criteria (< 15%) (Fig. 5d).

Predictive performance MPPE, MAPE, and RMSE were lower than 15% (mean values: 7.03%, 14.02%, and 12.14%, respectively). Paired results were highly correlated.

The results were statistically similar to the PL concentration obtained from the formula with HT. Therefore, the regression formula above was used for the calculations.

Because the HPLC–DAD method is still used for routine monitoring of MPA in the CMHI, cross-correlation and clinical validation were also performed between the VAMS and HPLC–DAD paired samples. However, statistical analysis denied interchangeability between VAMS-LC–MS/MS and HPLC–DAD methods (see Figs. 4g and h); the obtained regression formula was used for VAMS results conversion to MPA therapeutic range based on HPLC–DAD (statistical details shown in Table 2). The converted VAMS results using the prescribed formula fulfilled almost all requirements during clinical validation, except for the MAPE prediction factor (see Figs. 5e, f, and Table 2).

The results of MPA determination in an independent group of patients [*n* = 30] (not included in the above-described cross- and clinical validation) were compared using statistical tools used for method comparison. When the VAMS–LC–MS/MS analysis results were recalculated using the regression formula and compared in the next step with the PL–LC–MS/MS results, they entirely fulfilled the acceptance criteria. Pearson’s correlation coefficient equals 0.99 (0.96–0.99; 95% CI), while the slope was set at − 0.03 (ranging from − 0.54 to 0.06), and the intercept was set at 0.98 (ranging from 0.80 to 1.29). The mean percentage bias was calculated as − 0.80% (ranging from − 4.80 to 4.64), and more than 67% of paired samples fulfilled EMA (preference < 20%) as well as IATDMCT (preference < 15%) acceptance criteria. The predictive performance ratio was < 15%.

**Fig. 6:**
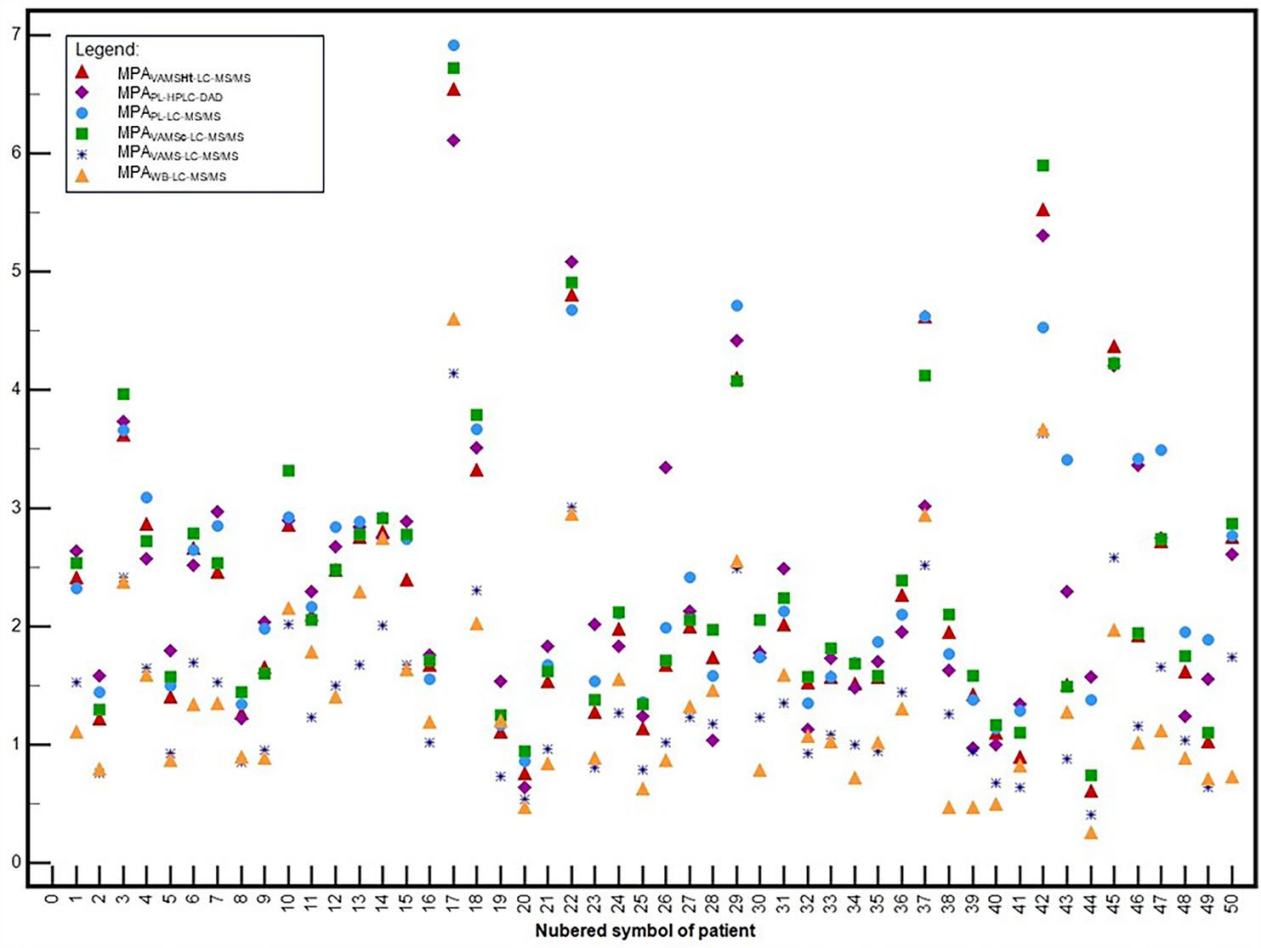
MPA determination results using different methods for each pediatric patient after renal transplantation—presented diagram included concentrations before and after conversion using formulas based on hematocrit value and Passing-Bablok regression model [*n* = 50]. The patient’s blinded numbers were assigned randomly. The meaning of using points, shapes, and colors is described in the legend on the graph. *MPA*_*VAMSHt-LC–MS/MS*_ mycophenolic acid concentration corrected with hematocrit, *MPA*_*PL-HPLC–DAD*_ mycophenolic acid concentration in plasma sample determined HPLC–DAD, *MPA*_*PL-LC–MS/MS*_ mycophenolic acid concentration in plasma sample determined LC–MS/MS, *MPA*_*VAMSc-LC–MS/MS*_ mycophenolic acid concentration corrected with regression, *MPA*_*VAMS-LC–MS/MS*_ mycophenolic acid concentration in VAMS sample determined LC–MS/MS, *MPA*_*WB-LC–MS/MS*_ mycophenolic acid concentration in whole blood sample determined LC–MS/MS, *VAMS* volumetric absorptive microsampling

The summarized results of the MPA measurements obtained using different sampling techniques and analytical methods are shown on a multi-point graph (Fig. 6). A table presenting the results of the MPA determination is provided in the Supplementary file (Sect. 7). For each patient, including the clinical validation.

## Discussion

This study presented successfully validated methods of MPA quantification in PL, WB, and VAMS capillary blood. In addition to the analytical aspect of method development, cross-validation, and clinical validation were performed in the pediatric population after renal transplantation. We developed a method for MPA determination in WB but only compared the MPA levels. The MPA levels in the WB and VAMS samples were notably lower than those in the PL. Therefore, the results should be interpreted carefully, and a new therapeutic range is required. The MPA concentration was lower in WB than in PL because of the dilution effect in WB [23, 24]. On the other hand, a more straightforward approach is based on MPA concentration conversion from WB to PL levels based on previously validated conversion formulas, as in the present study. However, only this approach allowed for comparing MPA concentrations in VAMS-based capillary blood and WB.

The main reasons for MPA monitoring are inter- and intra-individual dose/exposure relationships, adverse effects, and confounding factors such as food-drug interactions [1–4].In the pediatric population, close monitoring of MPA is essential for recognizing and evaluating adherence to therapy. Clinical validation of the method after analytical validation is necessary to ensure the reliability of the results. Only five studies have described clinical proof of MPA determination using VAMS [24–28]. Previously published studies have been performed in adult liver or renal transplant recipients. The relative differences in the corrected MPA concentrations evaluated by Paniagua-Gonzalez in the two studies were − 3.95% and 2.04%, respectively, while 95% and 75% of paired samples were within the acceptance bias range (± 20%) [24, 25]. Zwart et al. qualified 17.7% of the paired samples within the mentioned range and 84.4% after correction using a regression equation [26]. The relative difference evaluated using the Bland–Altman plot was equal to 7%. In this study, samples were collected at pre-dose (C_0_) and post-dose concentrations (C_1_, C_2_, C_3_; 1, 2, 3 h after drug administration, respectively) [26]. In other studies, paired time points were collected (before and after drug dosage) or only for trough concentration measurements (as in the present study). In a study by Wang et al., the mean bias was − 1.5% and more than 85% of paired samples fulfilled the EMA and clinical acceptance criteria (± 20 and 15%, respectively, for 67% of paired samples) [16, 27]. In the latest study, average bias and MAPE were − 5.3% and 9.50%, respectively, which fulfilled established criteria. Similar to our research, the ISR experimental results fulfilled the EMA criteria (84% of samples met the acceptance criteria) [28].

Recently published a comprehensive review of VAMS, graded assays according to their clinical utility, and applications for further studies [14]. Almost all published methods for MPA quantification have qualified into the category characterized as suitable for clinical use (negligible HT Effect, good cross-validation results, and high recovery values), which proved relatively high usability for TDM [14].

Following the study by Koster et al. MPA concentrations in VAMS samplers may be considered stable at RT, 37 °C, and – 20 ℃ for 60, 30, and 50 days, respectively [23]. Similar results were obtained in Paniagua-Gonzales study [24, 25]. Wang et al. reported a deviation of less than 15% for MPA concentration after storage at 4 ℃, whereas it was higher for VAMS stored at RT. In our study, MPA was stable at RT and 4 °C in VAMS for approximately 42 days. Preferably, the tips should be kept at RT in the dark to achieve higher stability. The limited stability of MPAG may cause its overestimation during long-term storage.

Our study showed the interchangeability of HPLC–DAD and LC–MS/MS methods owing to satisfactory regression parameters in the tested calibration range. However, these methods are similar; the higher sensitivity and selectivity of the LC–MS/MS method are considered the main benefits. Based on the variable calibration range, LC–MS/MS is the method of choice for simultaneous determination with other immunosuppressants [7, 23]. In this study, clinical validation of the regression formula was successful, in contrast to previously published studies [24–28]. However, the prescribed conversion may have been performed in an independent sample (not included in the validation). Still, on the other hand, more data for multi-center validation between groups and confirmation of the prescribed formula should be performed in the case of different specific samples.

The present study has some limitations. First, the pediatric population included in the study was relatively large; however, samples were obtained only once for trough concentration measurements. It should be noted that the validated method concentration range was established directly according to the trough concentration measurements. One of the main limitations of VAMS-based immunosuppressant determination is the limited access to proficiency testing schemes (PT). High interlaboratory variability (ranging from 13.2% to 18.2% for TAC and other immunosuppressants), concluded by Veenhof et al. in a pilot PT of immunosuppressants in VAMS, confirmed that standardization and external harmonization are strictly needed [29].

## Conclusions

The developed and fully validated methods were successfully applied to MPA measurements in VAMS samples, PL, and WB. The VAMS strategy is strictly beneficial owing to limited access to pediatric transplant clinics and experience with the SARS-CoV-2 pandemic restrictions. Furthermore, the method developed in this study can be used to monitor adherence to immunosuppressive therapy. To the best of our knowledge, this is the first LC–MS/MS method used for MPA determination in VAMS samples obtained from a pediatric population. The successful clinical validation of the conversion formula-based regression model is a primary contributor to the literature and VAMS implementation for MPA measurements in the pediatric population, which has not been described previously in the literature.

## Supplementary Information

Below is the link to the electronic supplementary material.

Supplementary file1 (PDF 437 KB)

## Data Availability

All data supporting the findings of this study are available in the paper and its Supplementary Information. Detailed references to the individual supplementary files are provided in the manuscript.

## Abbreviations

AcMPAG: Mycophenolic acid acyl-glucuronide
AR: Absolute recovery
C_0_: Trough concentration
CEDIA: Cloned enzyme donor immunoassay
CMHI: Children’s Memorial Health Institute
CV: Coefficient of variation
DBS: Dried-blood-spot
DL: Desolvatation line
EDTA: Ethylenediamine tetraacetic acid
EMA: European Medicines Agency
EMIT: Enzyme multiplied immunoassay technique
ESI( +): Positive-ion mode with electrospray ionization
FDCC: Fixed-dose concentration controlled trial
FLD: Fluorescence detection
FBPP: Fraction bound to plasma proteins
GCP: Good Clinical Practice
HB: Heat block
HP: Heparinized plasma
HPLC–DAD: High-performance liquid chromatography coupled with diode array detection
HT: Hematocrit
HQC: Highest quality control
IA: Immunoassay
IATDMCT: International Association of Therapeutic Drug Monitoring and Clinical Toxicology
IMPDH: Inosine monophosphate dehydrogenase
IS: Internal standard
ISR: Incurred sample reanalysis
KTx: Kidney transplantation
LC–MS/MS: Liquid chromatography–tandem mass spectrometry
LLE: Liquid–liquid extraction
LLOQ: Lower limit of quantification
LQC: Lowest quality control
MAPE: Median absolute percentage prediction error
ME: Matrix effect
MQC: Medium quality control
MMF: Mycophenolate mofetil
MPA: Mycophenolic acid
MPAG: Mycophenolic acid glucuronide
MPE: Median prediction error
MPPE: Median percentage prediction error
MRM: Multiple reaction monitoring
PE: Process efficacy
PETINIA: Particle-enhanced turbidimetric inhibition immunoassay
PK: Pharmacokinetic
PL: Plasma
R_t_: Retention time
RMSE: Root mean squared prediction error
RT: Room temperature
QC: Quality control
S/N: Signal-to-noise ratio
SD: Standard deviation
SR: Serum
TAC: Tacrolimus
TDM: Therapeutic drug monitoring
UHPLC-MS/MS: Ultra-high-performance liquid chromatography-tandem mass spectrometry
URIS: Structurally unrelated internal standard
UV: Ultraviolet detection
VAMS: Volumetric absorptive microsampling
WB: Whole blood
