# Supplementary material for: Therapeutic drug monitoring of mycophenolic acid (MPA) using volumetric absorptive microsampling (VAMS) in pediatric renal transplant recipients: ultra-high-performance liquid chromatography-tandem mass spectrometry analytical method development, cross-validation, and clinical application
Source: Pharmacol Rep. 2023 Jul 15;75(4):1026–42. doi: 10.1007/s43440-023-00509-w (PMC10374821; doi:10.1007/s43440-023-00509-w)
Supplement: Supplementary file 1 — Supplementary file1 (PDF 437 KB) [file 43440_2023_509_MOESM1_ESM.pdf]

SUPPLEMENTARY FILE

Therapeutic drug monitoring of mycophenolic acid (MPA) using volumetric absorptive microsampling (VAMS) in pediatric renal transplant recipients – ultra-high-performance liquid chromatography-tandem mass spectrometry analytical method development, cross-validation, and clinical application

Arkadiusz Kocur[1,2*], Jacek Rubik[3#], Paweł Czarnowski[4#], Agnieszka Czajkowska[2#], Dorota Marszałek[1], Maciej Sierakowski[5], Marta Górska[2], Tomasz Pawiński[1]

[#]JR, PC, and AC contributed equally to this work.

ORCID: **AK** 0000-0002-4833-8532, **PC** 0000-0002-3418-1837, **JR** 0000-0002-3392-2154, **AC** 0000-0003-0199-5751, **DM** 0000-0002-3189-5439, **MS** 0000-0003-3770-2762, **TP** 0000-0001-9110-4312

[1] Department of Drug Chemistry, Medical University of Warsaw, 1 Banacha St., 02-091 Warsaw, Poland;

[2] Pharmacokinetics Laboratory, Department of Biochemistry, Radioimmunology, and Experimental Medicine, The Children's Memorial Health Institute, Dzieci Polskich 20, 04-730 Warsaw, Poland.

[3] Department of Nephrology, Kidney Transplantation, and Arterial Hypertension, The Children's Memorial Health Institute, Dzieci Polskich 20, 04-730, Warsaw, Poland

[4] Department of Genetics, Maria Sklodowska-Curie National Research Institute of Oncology, Roentgena 5, 02-781 Warsaw, Poland

[5] Institute of Biological Sciences, Cardinal Stefan Wyszynski University in Warsaw, 1/3 Kazimierza Wóycickiego St., 01-938 Warsaw, Poland.

***Corresponding:** Arkadiusz Kocur, Department of Drug Chemistry, Medical University of Warsaw, 1 Banacha St., 02-091 Warsaw, Poland; tel. (+48 22) 57 20 630; fax. (+48 22) 57 20 697; e-mail: arkadiusz.kocur@wum.edu.pl

Running head:

Therapeutic drug monitoring of mycophenolic acid (MPA) using volumetric absorptive microsampling (VAMS) in pediatric renal transplant recipients

1. Materials

The standard reference MPA (chemical purity ≥98.00%) was obtained from Sigma-Aldrich (St. Louis, MO, USA), whereas its SIL-IS (stable-isotope-labeled internal-standard), MPA-d_3 (88.00% chemical purity, 99.10% isotopic purity) was obtained from Toronto Research Chemicals (Toronto, ON, Canada). The standard for MPAG monitoring in the LC-MS/MS assay was obtained from Sigma-Aldrich (St. Louis, MO, USA). LC-MS purity-grade solvents (acetonitrile and methanol) were acquired from Merck (Darmstadt, Germany). Reagents for mobile phase preparation, such as ammonium fluoride (>99.99% chemical purity) and formic acid for LC-MS (>99.99% chemical purity), were purchased from Sigma-Aldrich (St. Louis, MO, USA). Zinc sulfate heptahydrate ($ZnSO_4{\cdot}7H_2O$, >99.00% purity) was purchased from Merck (Darmstadt, Germany). Appropriate water purity was systematically achieved using a DL2-400 Polwater system (Labopol-Polwater, Kraków, Poland).

Fresh whole blood (WB) and plasma (PL) for method validation were systematically obtained from healthy donors untreated with immunosuppressive drugs (TAC and MPA) at the Regional Centre of Blood Donation and Hemotherapy (Warsaw, Poland). Blood was stored at 4°C, while plasma was frozen at -20°C and used to prepare calibration curves for one or four weeks, respectively.

The above reference and internal standards were stored at −20°C in a freezer to maintain appropriate stability. Other chemical substances and reagents, such as the liquids used for mobile phase preparation, analyte extraction, and protein precipitation, were stored at room temperature or 4°C when prepared in the experimental mixtures.

The VAMS-Mitra™ 10 µL samplers for capillary-blood collection and complementary 96-sample autorack for sampler drying were purchased from Neoteryx (Torrance, CA, USA). Vacutainer test tubes (1.6 mL) containing K3-EDTA (tripotassium salt of ethylenediamine tetra-acetic acid) as an anticoagulant for whole-blood collection, and 4 mL test tubes with clot activator, as well as lancets, and blood collection sets, were obtained from Becton Dickinson (Warsaw, Poland) or Sarstedt (Nümbrecht, Germany). Simple laboratory materials such as tips, test tubes, and falcon tubes were purchased from Sarstedt (Nümbrecht, Germany) or GenoPlast Biotech (Rokocin, Poland). Chromatographic vials integrated with 300 µL inserts and complementary screw caps were obtained from Thermo Scientific (Waltham, MA, USA) or Agilent (Santa Clara, CA, USA).

2. Calibrators

Primary stock solutions were prepared in glass ampules using solid standards for MPA (1 mg/mL), MPA- d_3 (0.1 mg/mL), and MPAG (0.1 mg/mL) via dissolving in methanol: water mixture (50:50, v/v). The above solutions were appropriately diluted to obtain the estimated concentration of working solutions and finally to generate the calibration curve. The SIL-IS working solution was prepared at a 25μL/mL concentration level and, in the next step, diluted during calibration curve preparation. The MPAG solution has diluted a hundredfold and was used only as a control for chromatographic separation of MPA and MPAG in patients' samples. Notable, the QC working solutions were prepared from other primary solutions of MPA than calibration curve points (but the same primary solution concentration-1 mg/mL). Working solutions were stored at -20°C in the freezer.

During the investigation, three types of matrices were used for calibration methods: plasma, whole blood, and capillary blood in VAMS samplers. The WB and PL samples were fortified abreast by spiking 50μL of each matrix with 10μL of calibration working solution or QC working solutions. The VAMS calibration sample was prepared by spiking the 50μL of WB with 10μL of the appropriate working solution after mixing by self-automated vortex (Chemland, Stargard, Poland) gently absorbing by VAMS tip. The calibration levels and quality controls (QC) for MPA were prepared at levels: 0.25, 0.35 (lower quality control, LQC), 0.5, 1.0, 2.5, 3.50 (medium quality control, MQC), 5.0, 10.0, 12.5 (higher quality control, HQC), and 15.0 μg/mL. The WB and PL without immunosuppressants were obtained from healthy volunteers.

3. Sample preparation protocol

The 50μL of WB or PL was diluted with 90 μL pure water, and 10 μL of internal standard (d_3-MPA) was added. In the next step, 400 μL of the precipitation mixture (0.1M zinc sulfate aqueous solution and acetonitrile, 50:50 (v/v)) was added. After that, the sample was shaken for 10 min at RT using an automatic shaker (ThermoScientific, Waltham, MA, USA) and centrifuged for 10 min under 3500 rpm at 4°C in MPW-375 (MPW Medical Instruments, Warsaw, Poland). 250 μL of obtained upper phase was transferred into insert integrated glass vials and analyzed with LC-MS/MS.

Loaded VAMS tips, after a minimum of 1h-drying, were removed and transferred into 150μL of pure deionized water. For analyte extraction, the samples were shaken at RT for 1 h at a frequency of 1000rpm using the above-mentioned shaker. After that, 10μL of IS solution and 150 μL above precipitation mixture were added. From this moment, the sample was treated under the same conditions as WB/PL sample.

4. Representative chromatograms

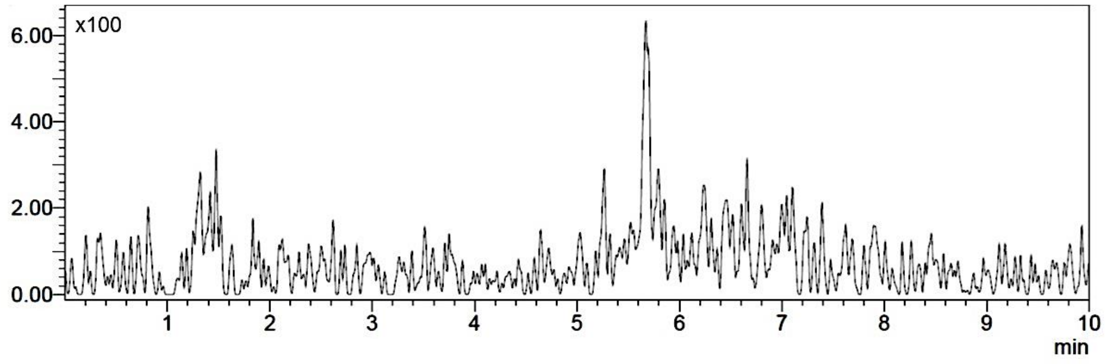

(a)

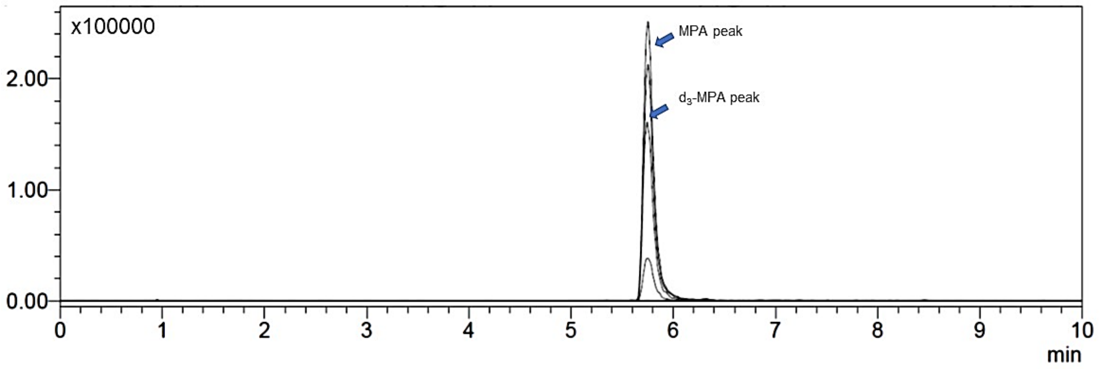

(b)

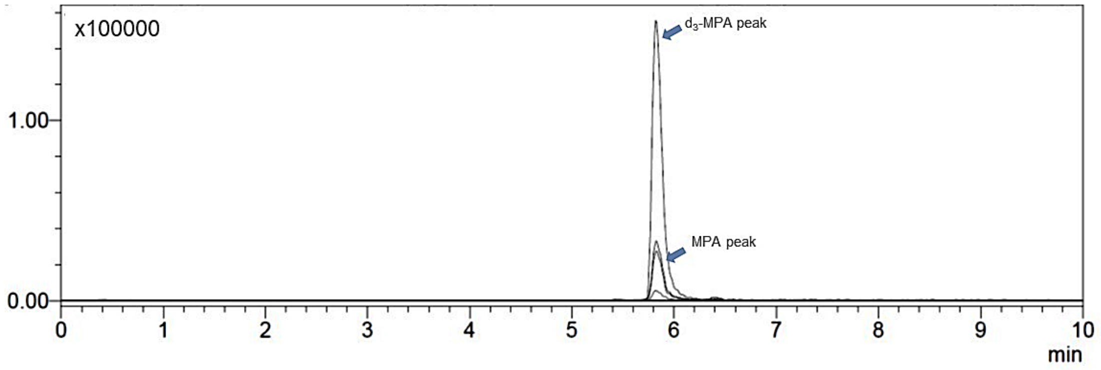

(c)

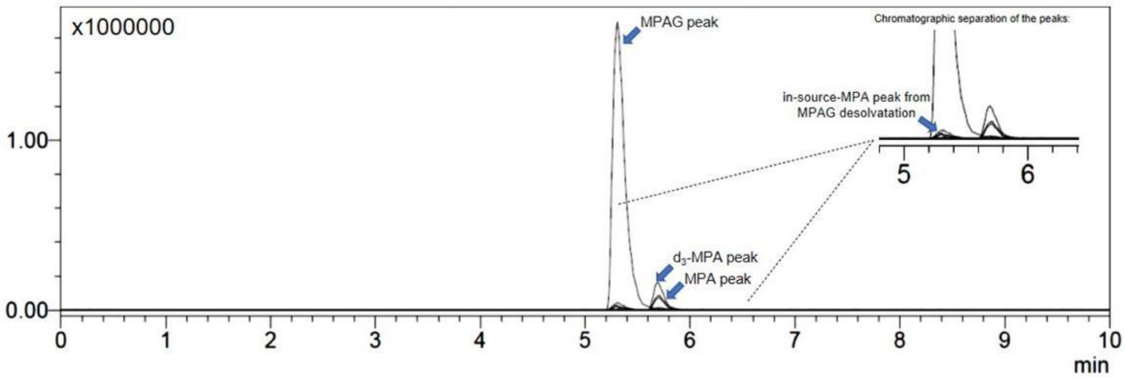

(d)

Figure. Representative chromatograms for (a) blank VAMS sample, (b) HQC in the VAMS sample, (c) LQC in the VAMS sample, and (d) patient VAMS sample.

5. Summary of the analytical validation process

Table 1 Results of intra-run and between-run precision and accuracy evaluation [n=10]

Parameter	0.25 µg/mL			0.35 µg/mL			12.50 µg/mL			15.00 µg/mL		
	WB	PL	VAMS	WB	PL	VAMS	WB	PL	VAMS	WB	PL	VAMS
**Intra-run accuracy and precision [n=10]**												
C_{MPA} [µg/mL]	0.26 ± 0.02	0.25 ± 0.02	0.27 ± 0.02	0.36 ± 0.02	0.34 ± 0.03	0.36 ± 0.03	12.55 ± 0.32	12.12 ± 0.51	12.57 ± 0.23	15.16 ± 0.44	14.88 ± 0.61	14.37 ± 0.62
Accuracy [%]	102.72	98.25	109.45	97.71	96.38	102.14	100.36	96.85	100.57	101.05	99.22	98.21
Precision [%]	6.75	8.48	6.82	4.96	8.92	6.53	2.58	4.19	1.83	2.91	4.14	2.29
**Between-run accuracy and precision [n=10]**												
C_{MPA} [µg/mL]	0.25 ± 0.02	0.34 ± 0.03	0.26 ± 0.02	0.35 ± 0.02	0.34 ± 0.03	0.38 ± 0.01	12.55 ± 0.32	12.12 ± 0.51	12.59 ± 0.30	15.33 ± 0.45	14.88 ± 0.62	14.89 ± 0.62
Accuracy [%]	98.25	96.38	105.78	100.08	96.38	108.14	100.36	96.85	100.76	102.18	99.22	98.72
Precision [%]	8.48	8.92	5.85	5.43	8.48	3.75	2.58	4.19	2.39	2.96	4.14	1.54

Table 2 Results of stability in autosampler examination [n=6]

Parameter	0.35 µg/mL			12.50 µg/mL		
	WB	PL	VAMS	WB	PL	VAMS
**Initial in autosampler**						
C_{MPA} [µg/mL]	0.38 ± 0.02	0.36 ± 0.03	0.38 ± 0.02	12.36 ± 0.42	12.28 ± 0.21	12.86 ± 0.17
Stability [%]	100.00	100.00	100.00	100.00	100.00	100.00
**After 24h in autosampler**						
C_{MPA} [µg/mL]	0.36 ± 0.02	0.34 ± 0.02	0.37 ± 0.02	12.36 ± 0.58	12.15 ± 0.49	12.61 ± 0.45
Stability [%]	98.68	94.73	97.37	96.19	98.94	98.10
**After 3 days in autosampler**						
C_{MPA} [µg/mL]	0.34 ± 0.02	0.34 ± 0.02	0.33 ± 0.02	11.74 ± 0.29	11.93 ± 0.63	12.49 ± 0.32
Stability [%]	96.01	93.05	91.57	94.98	97.14	97.12
**After 5 days in autosampler**						
C_{MPA} [µg/mL]	0.33 ± 0.03	0.31 ± 0.03	0.32 ± 0.02	10.76 ± 0.68	11.87 ± 0.46	12.19 ± 0.41
Stability [%]	91.66	86.11	86.84	87.05	90.39	92.34

Table 3 Results of short-term stability examination [n=6]

Parameter	0.35 µg/mL			12.50 µg/mL		
	0h	-2h	+2h	0h	-2h	+2h
**WB**						
C_{MPA} [µg/mL]	0.36 ± 0.02	0.32 ± 0.04	0.33 ± 0.09	12.41 ± 0.33	12.22 ± 0.42	12.13 ± 0.51
Stability [%]	100.00	91.23	93.26	100.00	96.67	96.74
**PL**						
C_{MPA} [µg/mL]	0.33 ± 0.03	0.34 ± 0.06	0.35 ± 0.11	12.56 ± 0.37	12.23 ± 0.26	12.36 ± 0.63
Stability [%]	100.00	101.23	100.98	100.00	97.12	98.16
**VAMS**						
C_{MPA} [µg/mL]	0.36 ± 0.02	0.35 ± 0.08	0.32 ± 0.10	12.62 ± 0.29	11.86 ± 0.31	12.09 ± 0.65
Stability [%]	100.00	96.69	91.26	100.00	96.97	95.89

Table 4 Results of matrix effect (ME), process efficiency (PE), and absolute recovery (AR) evaluation

Parameter	0.35 µg/mL			12.50 µg/mL		
	MPA	IS	F	MPA	IS	F
**WB**						
ME [%]	-25.83 ± 9.63	-35.52 ± 11.15	0.84 ± 0.13	-28.04 ± 8.34	-21.85 ± 7.02	0.79 ± 0.10
PE [%]	74.16 ± 3.26	64.47 ± 4.15	104.00 ± 2.45	71.96 ± 2.59	78.14 ± 3.01	97.28 ± 4.33
AR [%]	62.58 ± 4.86	62.19 ± 3.99	101.00 ± 4.12	67.71 ± 2.94	69.85 ± 2.88	99.79 ± 3.68
**PL**						
ME [%]	-24.96 ± 5.40	-22.11 ± 2.15	-8.73 ± 1.14	-24.93 ± 6.68	-16.63 ± 2.29	-1.12 ± 0.22
PE [%]	71.39 ± 3.57	77.88 ± 5.81	91.26 ± 2.88	75.77 ± 3.13	83.36 ± 3.84	98.87 ± 4.16
AR [%]	61.27 ± 5.36	69.87 ± 5.21	94.47 ± 3.01	65.31 ± 2.99	88.77 ± 3.80	98.96 ± 1.94
**VAMS**						
ME [%]	-25.58 ± 7.77	-24.16 ± 4.15	-1.18 ± 0.21	-37.34 ± 8.15	-29.43 ± 5.16	1.10 ± 0.19
PE [%]	64.26 ± 2.85	56.93 ± 2.02	98.81 ± 2.16	74.42 ± 3.14	75.84 ± 5.01	100.26 ± 4.44
AR [%]	63.52 ± 3.18	55.39 ± 4.12	93.77 ± 3.87	63.56 ± 4.22	61.95 ± 8.47	96.61 ± 6.21

6. Patients demographic data [n =50]

Variable	Patients' data*
number of patients	50
total number of samples	200
sex [male/female]	31/19
age [years]	11.26 ± 2.69 (4 – 17)
body weight [kg]	45.81 ± 15.41 (13.40 – 71.20)
height [m]	1.39 ± 0.16 (0.99 – 1.71)
MMF daily dose [mg]	678.95 ± 233.66 (250 – 1500)
MMF formulation [CellCept®]	50
hematocrit level [%]	36.29 ± 3.99 (29.00 – 46.20)
creatinine value [mg/dL]	0.88 ± 0.21 (0.56 – 1.49)
total protein concentration [g/L]	76.23 ± 4.28 (66 – 89)
albumin concentration [g/L]	45.38 ± 2.02 (42 – 51)
VAMS MPA concentration [μg/mL]	1.43 ± 0.77 (0.41 – 4.14)
VAMS MPA (corrected with Ht) concentration [μg/mL]	2.26 ± 1.23 (0.61 – 6.55)
VAMS MPA (corrected with formula) concentration [μg/mL]	2.37 ± 1.26 (0.74 – 6.72)
WB MPA concentration [μg/mL]	1.39 ± 0.84 (0.26 – 4.60)
PL MPA (LC-MS/MS) concentration [μg/mL]	2.47 ± 1.20 (0.86 – 6.92)
MPA (HPLC-DAD) concentration [μg/mL]	2.38 ± 1.16 (0.64 – 6.18)

*Data are expressed as mean ± SD (with min/max range)

7. Results of MPA determination in samples obtained from patients [n=50]

	MPA_DAD	MPA_WB	MPA_PL	MPA_VAMS	Hematocrit
	µg/mL	*µg/mL*	*µg/mL*	*µg/mL*	
1	2,64	1,11	2,32	1,53	0,37
2	1,58	0,80	1,44	0,76	0,38
3	3,73	2,38	3,66	2,42	0,33
4	2,57	1,59	3,09	1,65	0,43
5	1,8	0,87	1,5	0,93	0,34
6	2,52	1,34	2,65	1,69	0,37
7	2,97	1,35	2,85	1,53	0,38
8	1,22	0,90	1,34	0,85	0,33
9	2,04	0,89	1,98	0,95	0,43
10	2,9	2,16	2,93	2,02	0,29
11	2,3	1,79	2,17	1,23	0,42
12	2,68	1,41	2,84	1,50	0,39
13	2,84	2,30	2,89	1,68	0,39
14	2,75	2,75	2,93	2,01	0,37
15	2,89	1,64	2,74	1,68	0,30
16	1,76	1,19	1,56	1,02	0,39
17	6,11	4,60	6,92	4,14	0,37
18	3,51	2,03	3,67	2,31	0,31
19	1,54	1,20	1,17	0,73	0,35
20	0,64	0,47	0,86	0,54	0,29
21	1,83	0,84	1,68	0,96	0,38
22	5,08	2,95	4,68	3,01	0,37
23	2,02	0,89	1,54	0,81	0,37
24	1,83	1,56	2,11	1,27	0,36
25	1,24	0,63	1,36	0,79	0,31
26	3,34	0,87	1,99	1,02	0,39
27	2,13	1,32	2,42	1,23	0,39
28	1,04	1,46	1,58	1,18	0,32

29	4,42	2,56	4,71	2,49	0,39
30	1,78	0,79	1,74	1,23	0,31
31	2,49	1,59	2,13	1,35	0,33
32	1,13	1,07	1,35	0,93	0,39
33	1,73	1,03	1,57	1,08	0,31
34	1,48	0,72	1,69	1,00	0,34
35	1,7	1,02	1,87	0,94	0,40
36	1,95	1,31	2,1	1,44	0,37
37	3,02	2,94	4,62	2,52	0,46
38	1,63	0,47	1,77	1,26	0,35
39	0,97	0,47	1,38	0,94	0,34
40	1	0,50	1,14	0,68	0,38
41	1,34	0,82	1,29	0,64	0,29
42	5,31	3,67	4,53	3,63	0,34
43	2,3	1,28	3,41	0,88	0,42
44	1,57	0,26	1,38	0,41	0,32
45	4,2	1,97	4,23	2,58	0,41
46	3,36	1,02	3,42	1,16	0,40
47	2,75	1,12	3,49	1,66	0,39
48	1,24	0,89	1,95	1,04	0,36
49	1,56	0,71	1,89	0,64	0,38
50	2,61	0,73	2,77	1,74	0,37

MPA_DAD – MPA concentration determined with the HPLC-DAD method
MPA_WB – MPA concentration in whole blood determined with UHPLC-MS/MS method
MPA_PL – MPA concentration in plasma determined with UHPLC-MS/MS method
MPA_VAMS – MPA concentration in VAMS capillary blood determined with UHPLC-MS/MS method
